# Shellfish Dredging Pushes a Flexible Avian Top Predator out of a Marine Protected Area

**DOI:** 10.1371/journal.pbio.0040376

**Published:** 2006-11-14

**Authors:** Jan A van Gils, Theunis Piersma, Anne Dekinga, Bernard Spaans, Casper Kraan

**Affiliations:** 1 Department of Marine Ecology and Evolution, Royal Netherlands Institute for Sea Research (NIOZ), Texel, The Netherlands; 2 Animal Ecology Group, Centre for Ecological and Evolutionary Studies, University of Groningen, Haren, The Netherlands; University of York, United Kingdom

## Abstract

There is a widespread concern about the direct and indirect effects of industrial fisheries; this concern is particularly pertinent for so-called “marine protected areas” (MPAs), which should be safeguarded by national and international law. The intertidal flats of the Dutch Wadden Sea are a State Nature Monument and are protected under the Ramsar convention and the European Union's Habitat and Birds Directives. Until 2004, the Dutch government granted permission for ~75% of the intertidal flats to be exploited by mechanical dredgers for edible cockles *(Cerastoderma edule)*. Here we show that dredged areas belonged to the limited area of intertidal flats that were of sufficient quality for red knots *(Calidris canutus islandica),* a long-distance migrant molluscivore specialist, to feed. Dredging led to relatively lower settlement rates of cockles and also reduced their quality (ratio of flesh to shell). From 1998 to 2002, red knots increased gizzard mass to compensate for a gradual loss in shellfish quality, but this compensation was not sufficient and led to decreases in local survival. Therefore, the gradual destruction of the necessary intertidal resources explains both the loss of red knots from the Dutch Wadden Sea and the decline of the European wintering population. This study shows that MPAs that do not provide adequate protection from fishing may fail in their conservation objectives.

## Abstract 2

Shellfish extraction in a marine reserve so reduced food quality for red knots that they could no longer physiologically adapt to the changes, leading to the decline of this fully protected shorebird.

## Introduction

Worldwide benthic communities of intertidal soft sediments are seriously threatened by bottom-touching fisheries such as dredging [1]. Not only do target species directly decline in numbers because of removal, but target and non-target species are also indirectly affected [2,3]. In turn, predators such as migrant shorebirds, which make a living from benthic invertebrates, are declining in many parts of the world, and a link with commercial fishing activities seems evident [4–6]. Until 2004, three-quarters of the intertidal flats of the Dutch Wadden Sea were open to mechanical dredging for edible cockles, despite the high-level conservation status, despite the concerns about the damaging effects of cockle dredging to intertidal ecosystems, and despite the limited economic value of cockle dredging (a total of only 11 license holders maintained a fleet of just 22 fishing boats [7]).

A direct, immediate effect of dredging is the complete removal of all organisms larger than 19 mm in the 5-cm top layer. Because the sites dredged are usually the most biodiverse (C Kraan, T Piersma, A Dekinga, A Koolhaas, J Van der Meer, unpublished data), dredging may also affect smaller cockles; other bivalves such as blue mussels *(Mytilus edulis),* Baltic tellins *(Macoma balthica),* and sandgapers *(Mya arenaria);* polychaetes; and crustaceans such as shorecrabs *(Carcinus maenas)*. More indirectly and over longer time scales, sediments become coarser after dredging events, leading to reduced settlement success in both cockles and Baltic tellins [3]. Because grain size affects the feeding performance of bivalve mollusks [8], one expects prey condition or quality (defined as flesh-to-shell ratio [9]) to be negatively affected by dredging activities. We may thus expect mechanical dredging to lead to both short- and long-term declines in quantity and quality of a variety of macrobenthic organisms.

Shellfish-eating shorebirds can cope with reductions in both prey density and in prey quality, as long as the reductions are not too large and do not occur simultaneously (Figure 1). Red knots have been shown to flexibly adjust their digestive capacity to food quality so that food processing rates are just sufficient to obtain the daily amount of energy (i.e., flesh) required to maintain energy balance [9,10]. Maintaining a larger digestive system would be costly in terms of maintenance and transport costs [11]. Therefore, a knot's intake rate is often constrained by a digestive bottleneck [9,12]. This implies that a decline in prey density, although it may hamper maximum rates of prey collection (dictated by so-called “short-term functional responses” such as Holling's type II disk equation), will often only marginally affect the digestively constrained intake rate over the full low-tide period (Figure 1). Likewise, as the digestive system is flexible and can be adjusted rapidly [13], a decline in food quality can be compensated for by an increase in digestive capacity (Figure 1). By contrast, once both density and quality decline simultaneously, the situation may become critical. In such cases, intake rate may become too low to maintain daily energy balance (Figure 1).

**Figure 1:**
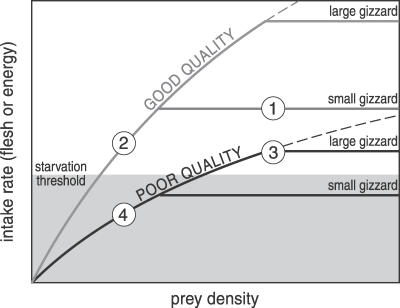
Conceptualization of Why a Simultaneous Reduction in Density and Quality of the Prey is Detrimental Holling's Type II functional response describes intake rate (be it flesh or energy) as a function of the density of either poor-quality (black lines) or good-quality (gray lines) prey. Digestive constraint limits shell-mass processing rate and is given for two gizzard sizes for each prey quality (horizontal cut-offs in functional response; digestively unconstrained intake rates continue as dashed lines). By knowing the threshold intake rate needed to avoid starvation (border between gray and white background), one can predict a bird's starvation chances on the basis of gizzard size and prey quality and density. (1) A small gizzard is sufficient to stay alive when prey is of good quality and occurs in high densities. Going from (1) to (2), prey density is reduced, which does not affect survival as intake rate remains above the critical threshold. Going from (1) to (3), prey quality (flesh-to-shell ratio) is reduced. To maintain a sufficient intake rate, the knot needs to increase its shell-mass processing rate, which requires a gizzard enlargement. Going from (1) to (4), the combined reduction in density and quality makes a gizzard enlargement no longer sufficient (as intake rate is now constrained by prey density), and the bird is bound to starve.

We studied dredging-induced changes in food quantity and quality and their effects on digestive physiology and survival in red knots (henceforth called knots) in the western Dutch Wadden Sea (53°14′ N, 5°10′ E). This site is of major importance for wintering *islandica* knots, and it is estimated that about 1/3–1/2 of the population stays or at least passes through the area each winter [14,15]. In an area of roughly 250 km^2^, we annually (1998–2002) sampled densities and qualities of knot food in great detail (Figure 2A). Each year, mechanical dredging took place from early September into December, immediately after the completion of our sampling program. We used the exact locations of dredging to categorize 1-km^2^ sample blocks as dredged or undredged (bearing in mind that this is not an ideal randomized block design, because blocks were selected by the fishermen). Because knots during our study period mostly consumed first-year cockles (mean percentage in diet ± standard error [SE] = 57.9 ± 2.8% of ingested flesh; *n* = 174 dropping samples), quantity and quality effects of dredging were studied with respect to newly settled cockles (≤16 mm) only.

**Figure 2:**
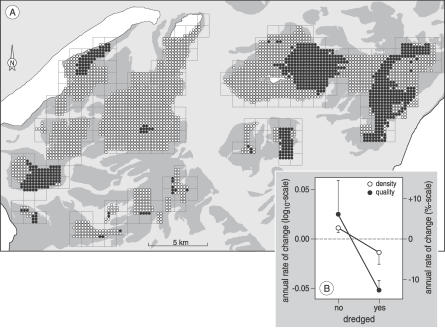
Study Area and Effects of Dredging (A) Map of the study area with 2,846 sampling stations (dots) categorized into 272 square kilometer blocks (squares containing 16 stations at most). A dot is filled when a station has been dredged at least once in 1998–2002 and is open when the station was never dredged during that period. (B) Densities of available cockles remained stable in dredged blocks, but they increased (+3% y^−1^) in undredged blocks (open dots ± SE bars). Quality of available cockles declined in dredged areas (−11% y^−1^), whereas it remained stable in undredged areas (filled dots ± SE bars).

We will make the point that shell fishing and shorebird conservation are incompatible. Marine Protected Areas (MPAs) are increasingly seen as a tool to manage sustainable fisheries in coastal ecosystems [16], which gives policy makers the feeling that they can achieve a win-win for both conservation and commercial exploitation. However, in practice, because nature conservation objectives are often fitted around the ongoing fisheries, conservation and commerce are not compatible [17] as exemplified here in the case of knots and shell fishing in the Dutch Wadden Sea.

## Results/Discussion

Densities of small cockles remained stable in areas mechanically dredged, whereas they increased by 2.6% per year in undredged areas (general linear model [GLM] with *n* = 271 blocks: *R* ^2^ = 0.02, *p* = 0.04) (Figure 2B). Moreover, quality of small cockles declined by 11.3% per year in dredged areas and remained stable in undredged areas (GLM with *n* = 59 blocks: *R* ^2^ = 0.07, *p* = 0.04) (Figure 2B). These results are consistent with a previous assessment [3] that showed dredged areas to become unattractive areas for cockles to settle, a finding that was explained by dredged sediments losing silt and becoming coarser. In deposit-feeding bivalves such as freshly settled cockles [18,19], coarser sediments may lead to worse feeding conditions [8] and therefore to reduced body condition, which, from the predator's point of view, equals reduced prey quality [20].

To express changes in carrying capacity in relation to dredging activity, we calculated for each year the percentage of blocks that would yield insufficient intake rates for knots to maintain a positive energy balance (taking 4.8 W as the critical metabolizable energy intake rate [21]). During our study period, the percentage of km^2^ blocks that were too poor for knots to feed increased from 66% in 1998 to 87% in 2002 (GLM with *n* = 5 y: *R* ^2^ = 0.80, *p* = 0.04) (Figure 3A). This was entirely due to an increase in previously suitable blocks that were dredged (GLM with *n* = 5 y: *R* ^2^ = 0.96, *p* = 0.003) (Figure 3A, dark gray bars). We calculated that of this 21% increase in unsuitability, 8% was due to a decline in prey densities alone, whereas the remaining 13% was due to the simultaneous decline in density and quality (note that these calculations include all potential prey species, not only cockles). In contrast, the percentage of unsuitable blocks that were never dredged before did not change (GLM with *n* = 5 y: *R* ^2^ = 0.02, *p* = 0.80 (Figure 3A, light gray bars). As a consequence of the widespread dredging in the best areas, diet quality declined by 11.7% per year (GLM with *n* = 174 dropping samples: *R* ^2^ = 0.05, *p* = 0.003) (Figure 3B), a similar rate of decline (11.3%) as shown by the quality of cockles, the knots' main prey, in dredged areas. To compensate for reductions in diet quality, knots should increase gizzard mass [9,13]. Indeed, in the course of the study period, gizzard mass increased by 3.4% per year (GLM with *n* = 644 birds: *R* ^2^ = 0.02, *p* = 0.0001) (Figure 3C). This increase in gizzard mass exactly matches our quantitative expectations, because experimental results on shell mass processing rates [9] imply that gizzard mass should vary inversely with the square root of prey quality (i.e., square root of the annual 11.7% decline in prey quality = 3.4%). More specifically, gizzard masses do not deviate from the predicted gizzard masses required to balance the energy budget (predicted from yearly average prey quality values [9,10], *p* > 0.1, *n* = 484).

**Figure 3:**
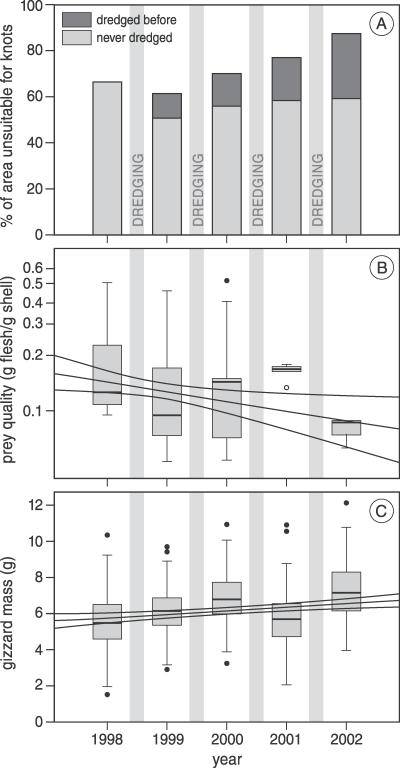
Changes in Suitability, Prey Quality, and Gizzard Mass (A) The percentage of blocks that yielded insufficient intake rates (<4.8 W) increased over time due to an increase in unsuitable blocks being dredged in previous years (dark gray bars; as opposed to light gray bars indicating unsuitable blocks that were never dredged). (B) Quality of prey included in the diet has declined over time (box-and-whisker plot, line gives GLM ± 95% confidence intervals). (C) In response, gizzard mass has increased over time (boxes and lines as in (B)).

Nevertheless, resightings of individually color-banded birds, whose gizzards were measured before release, suggest that on average, birds not seen in our study area within the year after release had undersized gizzards (*p* < 0.01, *n* = 362), whereas individuals that we did see again had gizzards that enabled them to achieve a balanced daily energy budget (*p* > 0.4, *n* = 122) (Figure 4A). That birds returning from the high-arctic breeding grounds with undersized gizzards [22] do not simply enlarge their gizzard may be explained by time and energy constraints [13,23]. Birds arriving with too small a gizzard may need more time to adjust their gizzard than their fat stores allow them, and therefore they face starvation unless they leave the area (indeed we found that birds with a smaller gizzard had a lower body mass; *R* ^2^ = 0.06, *p* < 0.00001, *n* = 483). This implies that the proportion of birds arriving with a gizzard large enough to survive increases as a function of food quality at the arrival destination (Figure 4A and 4B). Indeed, local annual survival rate (calculated from resighting rates of color-banded birds) increased with food quality in the expected direction (Figure 4C). When we estimated the distribution of gizzard masses upon arrival (by selecting only those birds caught in the Wadden Sea during late July) and calculated, for various degrees of gizzard flexibility, the proportion of birds that would survive the critical gizzard-enlargement phase (Figure 4B and 4C), the best fit with the actual survival data was obtained if knots had time to increase their gizzard by 1 g only (Figure 4C).

**Figure 4:**
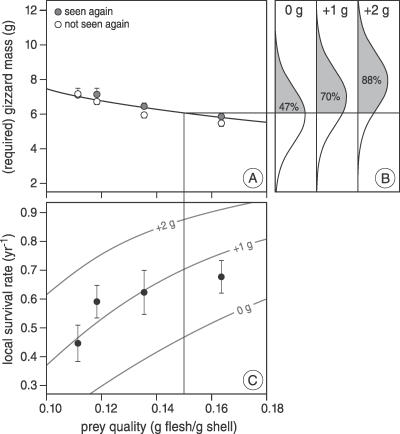
Quantitative Relations between Prey Quality, Gizzard Mass, and Local Survival Rate (A) Gizzard mass required to maintain energy balance declines as a function of prey quality (solid line). Gizzards of birds seen again after catching fit this relationship (gray dots; mean ± SE), whereas gizzards of birds not seen again are significantly smaller (open dots; note that both groups almost entirely overlap in the poorest-quality year). (B) Observed gizzard masses upon arrival are distributed according to the left-most normal distribution. If there were no room for flexibly adjusting gizzard mass (0 g), only 47% of the arriving knots would be able to avoid starvation (shaded area) at an example prey quality of 0.15 g flesh per g shell (dashed line). If there were room for flexibility (+1 and +2 g in this example), a much larger proportion would be able to survive (respectively 70% and 88%). (C) Observed local survival rate (y^−1^; ± SE) increased as a function of prey quality and best matched with predicted survival in a +1-g flexibility scenario.

Color-banded knots that disappeared from our study area may have died or, perhaps more likely for a wide-ranging migrant, emigrated to other areas such as the estuaries in the United Kingdom, where they probably paid a mortality cost due to the extra travel and/or due to uncertainties in the food supply at their new destination (where they also fed on hard-shelled prey, in the past [24,25] and more recently [26,27], and thus faced gizzard-related mortality; note that knots are “forced” to feed on hard-shelled prey as their pressure-sensitive bill tip can only detect hard objects buried in soft sediments [28]). In any case, the declining numbers of knots wintering in the Dutch Wadden Sea [29] can be explained as a response to declining food conditions. Moreover, the 25% decline of the entire northwestern European wintering population between 1997–1998 and 2002–2003 (from ~330,000 to ~250,000) (G Austin, M Van Roomen, B Koks, T Piersma, unpublished data) can be explained by measured decreases in local survival of the Wadden Sea segment of the population during the study period (using the observed local survival rates, we estimated an extra mortality of 58,000 birds over the 5-y period). This study concludes that industrial forms of commercial exploitation of protected marine nature reserves in The Netherlands, by indirectly reducing food resource quality to such extents that changes can no longer be accommodated by adjustments of the digestive system, are directly responsible for the overall population decline of a fully protected shorebird species. This paper thereby adds to growing list of studies [17,30] stressing the uselessness of declaring a marine area as protected whenever the species living in it are seriously affected by the ongoing but regulated human activities.

## Materials and Methods

### Sampling prey density and quality.

From late July to early September 1998–2002, we sampled macrozoobenthos throughout the western Dutch Wadden Sea in a regular grid (250-m grid intersections; Figure 2A). In total, we visited 2,846 stations, of which the majority (75%) were sampled each year (89% in 4 out of 5 y). Stations were located using handheld global positioning system (GPS) receivers (Garmin 45 and 12; Garmin Corporation, Lenexa, Kansas, United States) and were either visited during low tide (on foot) or during high tide (by rubber boat). At each station, a sediment core was taken (15-cm diameter; 20-cm deep) that was sieved over a 1-mm mesh. To distinguish accessible prey from prey living beyond the reach of a knot's bill (4 cm), we sieved the top layer (upper 4 cm) separately from the bottom layer (only the “low-tide samples”). Mudsnails *(Hydrobia ulvae)* were sampled using a smaller core (7-cm diameter) and a finer mesh (0.5 mm). All potential prey items retained on the sieve were frozen (−20 °C) for later analyses. In the laboratory, items were identified with respect to species and size (to nearest mm; or, in the case of H. ulvae, to nearest 0.5 mm). Size classes were determined to distinguish ingestible prey from prey too large to be swallowed [12]. Methods to determine flesh and shell mass are explained elsewhere [3, 31].

### Mechanical dredging and its effects on cockle density and quality.

During each of the 5 y studied, mechanical dredging took place after our sampling program; i.e., from early September into December. Exact locations of dredging were known, because for reasons of internal control, every vessel had a GPS-logger onboard [7]. Cumulative seasonal data on dredging locations were available in the form of fine-scaled maps (resolution of 0.1 min latitude by 0.5 min longitude).

We analyzed the effects of mechanical dredging on cockles that were actually available to knots (i.e., ≤ 16 mm, because larger ones cannot be swallowed [32]). We pooled sampling stations in 272 blocks measuring 1 km by 1 km each (Figure 2A; many sampling stations did not have cockles available during multiple years making it impossible to study changes in quality at the spatial scale of stations). A block was considered dredged when at least one station was dredged in at least 1 y. Per block, we applied the following GLMs: log_10_(density + 1) = constant + year and log_10_(quality) = constant + year. Subsequently, we tested whether significant variation in the coefficients for year could be explained by whether a block was ever dredged or not during 1998–2001 (again using GLM).

### Suitability for knots.

The measurements on (available) prey densities and qualities allowed us to predict for each station an intake rate for a knot with an average-sized gizzard (6-g fresh mass). We did so by applying the so-called “digestive rate model,” a multi-species functional response that takes rates of digestion into account [33], which accurately predicted diet choice, patch choice, and intake rate in both free-ranging and captive knots [12,31]. Subsequently, after averaging these intake rates per block, we determined a block's suitability. A block was considered suitable when it yielded an intake rate sufficient to maintain energy balance when feeding for 10 h per day (corresponding to an intake rate of at least 0.3 mg dry flesh mass s^−1^, which equals a metabolizable energy intake rate of at least 4.8 W; note that the energy contents of 1 g of flesh is more or less constant in mollusks [34]). This approach is widely used [35,36] and adequately predicted the distribution of knots in an earlier study [21].

### Diets, gizzard sizes, and observed and predicted survival rates.

Diet quality was reconstructed by fecal analyses, following the procedures outlined by Dekinga and Piersma [37]. Dropping samples were collected during low tide at sites where flocks of knots had just fed. Across the 5 y of study, we analyzed 174 dropping samples, comprising 1–100 dropping(s) each (15 on average).

In order to estimate gizzard sizes and survival rates, we annually mistnetted knots from late July to late November, mostly near their main roost at Richel (an island located midnorth of our study area; Figure 2A), but occasionally near the isle of Schiermonnikoog (±75 km ENE of Richel). Based on the presence or absence of active wing molt [15], we determined subspecific identity and selected for the current analyses *islandica* individuals only (population numbers and survival rates are best known in this subspecies). Gizzard masses were estimated using ultrasonography (Pie 200 ultrasound, Pie Medical Benelux BV, Maastricht, The Netherlands; applied to 644 out of the 1,069 individuals selected). This method has been extensively calibrated [38] and has been successfully applied in earlier studies [9,12,13, 31]. Furthermore, to estimate survival rates, each bird was given a unique combination of color bands. We used the Cormack-Jolly-Seber model of the MARK software package [39] to estimate “local survival,” i.e., the actual survival during the first year after capture minus the (unknown) fraction of birds that emigrated permanently from our study area. In the model, annual survival rate was allowed to differ in the first year after capture and was assumed constant thereafter. This can be biologically interpreted as a variable proportion of birds becoming site faithful in each year. In case permanent emigration leads to death, the extra mortality equals the difference between first-year-after-capture global survival rate (mean = 0.73; SE = 0.03; calculated over all resightings, both inside and outside the Wadden Sea) and first-year-after-capture local survival rate. Assuming that each autumn, 100,000 individuals “try out” the Wadden Sea, this extra mortality in the NW-European wintering population as the result of emigration can thus be estimated. Note that we had 4 rather than 5 y of data, because our color-banding program started in 1998, yielding the first survival estimate for 1998–1999.

According to the following procedure, we predicted local survival rate for various degrees of gizzard flexibility (0–2 g) upon arrival in our study area (lines in Figure 4C). Based on experimental results on shell mass processing rates as a function of gizzard size [9], we calculated the minimal gizzard size required to avoid starvation as a function of prey quality (line in Figure 4A). Using the observed distribution of gizzard masses upon arrival (the 0-g distribution in Figure 4B, representing knots caught during late July only; *n* = 218; mean = 5.92; variance = 2.61), we then calculated the proportion of birds having a gizzard of at least this critical size, which would be the proportion of birds able to survive in our study area (47% at the example prey quality of 0.15 g flesh per g shell in Figure 4). Assuming that knots upon arrival have the flexibility to increase their gizzard slightly (e.g., by +1 g), we calculated an updated “effective” gizzard mass distribution upon arrival (i.e., a distribution that shifted by, e.g., +1 g; Figure 4B), yielding an updated proportion of birds with a large enough gizzard, i.e., able to survive (70% for the +1-g example). We refer to Van Gils et al. [9,10,20] for more details on modeling gizzard masses.

## Abbreviations

GLM: general linear model
MPA: marine protected area
SE: standard error

## References

[pbio-0040376-b001] Jackson JBC, Kirby MX, Berger WH, Bjorndal KA, Botsford LW (2001). Historical overfishing and the recent collapse of coastal ecosystems. Science.

[pbio-0040376-b002] Hiddink JG (2003). Effects of suction-dredging for cockles on non-target fauna in the Wadden Sea. J Sea Res.

[pbio-0040376-b003] Piersma T, Koolhaas A, Dekinga A, Beukema JJ, Dekker R (2001). Long-term indirect effects of mechanical cockle dredging on intertidal bivalve stocks in the Wadden Sea. J Appl Ecol.

[pbio-0040376-b004] Atkinson PW, Clark NA, Bell MC, Dare PJ, Clark JA (2003). Changes in commercially fished shellfish stocks and shorebird populations in the Wash, England. Biol Conserv.

[pbio-0040376-b005] Verhulst S, Ens BJ, Oosterbeek K, Rutten AL (2004). Shellfish fishery severely reduces condition and survival of oystercatchers despite creation of large marine protected areas. Ecol Soc.

[pbio-0040376-b006] Atkinson PW, Clark NA, Dodd SG, Moss D (2005). Changes in fisheries practices and Oystercatcher survival, recruitment and body mass in a marginal cockle fishery. Ardea.

[pbio-0040376-b007] Kamermans P, Smaal AC (2002). Mussel culture and cockle fisheries in The Netherlands: Finding a balance between economy and ecology. J Shellfish Res.

[pbio-0040376-b008] Drent J, Luttikhuizen PC, Piersma T (2004). Morphological dynamics in the foraging apparatus of a deposit feeding marine bivalve: Phenotypic plasticity and heritable effects. Funct Ecol.

[pbio-0040376-b009] Van Gils JA, Piersma T, Dekinga A, Dietz MW (2003). Cost-benefit analysis of mollusc-eating in a shorebird. II. Optimizing gizzard size in the face of seasonal demands. J Exp Biol.

[pbio-0040376-b010] Van Gils JA, Battley PF, Piersma T, Drent R (2005). Reinterpretation of gizzard sizes of red knots world-wide emphasises overriding importance of prey quality at migratory stopover sites. Proc R Soc Lond Ser B Biol Sci.

[pbio-0040376-b011] Piersma T, Dekinga A, Van Gils JA, Achterkamp B, Visser GH (2003). Cost-benefit analysis of mollusc-eating in a shorebird. I. Foraging and processing costs estimated by the doubly labelled water method. J Exp Biol.

[pbio-0040376-b012] Van Gils JA, Dekinga A, Spaans B, Vahl WK, Piersma T (2005). Digestive bottleneck affects foraging decisions in red knots Calidris canutus. II. Patch choice and length of working day. J Anim Ecol.

[pbio-0040376-b013] Dekinga A, Dietz MW, Koolhaas A, Piersma T (2001). Time course and reversibility of changes in the gizzards of red knots alternatively eating hard and soft food. J Exp Biol.

[pbio-0040376-b014] Davidson NC, Wilson JR (1992). The migration-system of European-wintering knots *Calidris canutus islandica*. Wader Study Group Bull.

[pbio-0040376-b015] Nebel S, Piersma T, Van Gils J, Dekinga A, Spaans B (2000). Length of stopover, fuel storage and a sex-bias in the occurrence of two subspecies of red knots (*Calidris c. canutus* and *islandica*) in the Dutch Wadden Sea during southward migration. Ardea.

[pbio-0040376-b016] Gerber LR, Botsford LW, Hastings A, Possingham HP, Gaines SD (2003). Population models for marine reserve design: A retrospective and prospective synthesis. Ecol Appl.

[pbio-0040376-b017] Jameson SC, Tupper MH, Ridley JM (2002). The three screen doors: Can marine “protected” areas be effective?. Mar Poll Bull.

[pbio-0040376-b018] Kang CK, Sauriau P-G, Richard P, Blanchard GF (1999). Food sources of the infaunal suspension-feeding bivalve Cerastoderma edule in a muddy sandflat of Marennes-Oléron Bay, as determined by analyses of carbon and nitrogen stable isotopes. Mar Ecol Prog Ser.

[pbio-0040376-b019] Rossi F, Herman PMJ, Middelburg JJ (2004). Interspecific and intraspecific variation of δ^13^C and δ^15^N in deposit- and suspension-feeding bivalves (Macoma balthica and Cerastoderma edule): Evidence of ontogenetic changes in feeding mode of Macoma balthica. Limnol Oceanogr.

[pbio-0040376-b020] Van Gils JA, Piersma T, Dekinga A, Battley PF (2006). Modelling phenotypic flexibility: An optimality analysis of gizzard size in red knots (Calidris canutus). Ardea.

[pbio-0040376-b021] Piersma T, Van Gils J, De Goeij P, Van der Meer J (1995). Holling's functional response model as a tool to link the food-finding mechanism of a probing shorebird with its spatial distribution. J Anim Ecol.

[pbio-0040376-b022] Piersma T, Gudmundsson GA, Lilliendahl K (1999). Rapid changes in the size of different functional organ and muscle groups during refueling in a long-distance migrating shorebird. Physiol Biochem Zool.

[pbio-0040376-b023] Piersma T, Dietz MW, Dekinga A, Nebel S, van Gils J (1999). Reversible size-changes in stomachs of shorebirds: When, to what extent, and why?. Acta Ornithol.

[pbio-0040376-b024] Goss-Custard JD, Jones RE, Newbery PE (1977). The ecology of the Wash. I. Distribution and diet of wading birds (Charadrii). J Appl Ecol.

[pbio-0040376-b025] Prater AJ (1972). The ecology of the Morecambe Bay. III. The food and feeding habits of knot (Calidris canutus L.) in Morecambe Bay. J Appl Ecol.

[pbio-0040376-b026] Atkinson PW, Crooks S, Drewitt A, Grant A, Rehfisch M (2004). Managed realignment in the UK: The first five years of colonization by birds. Ibis.

[pbio-0040376-b027] Stillman RA, West AD, Goss-Custard JD, McGrorty S, Frost NJ (2005). Predicting site quality for shorebird communities: A case study on the Humber estuary, UK. Mar Ecol Prog Ser.

[pbio-0040376-b028] Piersma T, Van Aelst R, Kurk K, Berkhoudt H, Maas LRM (1998). A new pressure sensory mechanism for prey detection in birds: the use of principles of seabed dynamics?. Proc R Soc Lond Ser B Biol Sci.

[pbio-0040376-b029] Van Roomen M, Van Turnhout C, Van Winden E, Koks B, Goedhart P (2005). Trends in benthivorous waterbirds in the Dutch Wadden Sea 1975–2002: Large differences between shellfish-eaters and worm-eaters. Limosa.

[pbio-0040376-b030] Caddy F, Surette T (2005). In retrospect the assumption of sustainability for Atlantic fisheries has proved an illusion. Rev Fish Biol Fish.

[pbio-0040376-b031] Van Gils JA, De Rooij SR, Van Belle J, Van der Meer J, Dekinga A (2005). Digestive bottleneck affects foraging decisions in red knots Calidris canutus. I. Prey choice. J Anim Ecol.

[pbio-0040376-b032] Zwarts L, Blomert A-M (1992). Why knots Calidris canutus take medium-sized Macoma balthica when six prey species are available. Mar Ecol Prog Ser.

[pbio-0040376-b033] Hirakawa H (1995). Diet optimization with a nutrient or toxin constraint. Theor Popul Biol.

[pbio-0040376-b034] Zwarts L, Wanink JH (1993). How the food supply harvestable by waders in the Wadden Sea depends on the variation in energy density, body weight, biomass, burying depth and behaviour of tidal-flat invertebrates. Neth J Sea Res.

[pbio-0040376-b035] Gill JA, Sutherland WJ, Norris K (2001). Depletion models can predict shorebird distribution at different spatial scales. Proc R Soc Lond Ser B Biol Sci.

[pbio-0040376-b036] Sutherland WJ, Anderson CW (1993). Predicting the distribution of individuals and the consequences of habitat loss: The role of prey depletion. J Theor Biol.

[pbio-0040376-b037] Dekinga A, Piersma T (1993). Reconstructing diet composition on the basis of faeces in a mollusc-eating wader, the knot Calidris canutus. Bird Study.

[pbio-0040376-b038] Dietz MW, Dekinga A, Piersma T, Verhulst S (1999). Estimating organ size in small migrating shorebirds with ultrasonography: an intercalibration exercise. Physiol Biochem Zool.

[pbio-0040376-b039] Sandercock BK (2003). Estimation of survival rates for wader populations: A review of mark-recapture methods. Wader Study Group Bull.

