# Expression of Concern: Shellfish dredging pushes a flexible avian top predator out of a marine protected area

**DOI:** 10.1371/journal.pbio.3003770

**Published:** 2026-04-22

**Authors:** The PLOS Biology Editors

Concerns about this article’s [1] findings and conclusions were raised in [2], and a response was subsequently published in [3]. The *PLOS Biology* Editors have re-evaluated the scientific validity of [1] in light of issues discussed in [2,3], and concluded that concerns raised about the analyses shown in Fig 4 of [1] undermine confidence in the article’s conclusions that cockle dredging in the study area had an adverse effect on red knot populations. Furthermore, our assessment is that the response in [3] did not satisfactorily resolve the issues, although a full analysis of the reported concerns [2] and responses [3] would require additional analysis beyond the scope of our post-publication editorial assessment.

Readers are therefore advised to interpret this article’s [1] results with caution in the context of the discussion in [2,3].
